# Book Reviews

**Published:** 1892-07

**Authors:** 


### *BOOK REVIEWS.*

---

Regional Anatomy, in its Relation to Medicine and Surgery. By George McClellan, M. D., Lecturer on Descriptive and Regional Anatomy at the Pennsylvania School of Anatomy; Professor of Anatomy at the Pennsylvania Academy of Fine Arts, etc. In two volumes. Vol. I. J. B. Lippincott Company, Philadelphia.

In the first place, it is to be said, to the infinite credit of this excellent and handsome work, that it contains *no diagrams*. The full-page plates are photographs of nature taken and colored by the author from his own dissections. For this work Dr. McClellan made about three hundred dissections and relied upon the "unfailing precision of the camera" to present the relations of the parts as they actually exist. The author's theory and motive in producing this work cannot be gainsaid—that the best book on anatomy *is the body itself.* "Anatomy is not that which is taught in the schools," said Bichat; neither is it that which is taught in the books. A correct knowledge of anatomy cannot be gained by the use of diagrams designed by the artist with the view of assisting the student by simplifying nature.

For instance, no limit of descriptive text and no end of drawings and plates can give the student an accurate conception of the foramen of Winslow or of the lateral ventricles of the brain. The author appreciates this fact and has given us, as nearly as possible, exact photographic reproductions of the parts exposed on the dissecting table. As to the plates, they are of course accurate, and the greater part of them show the different structures and their mutual relations with satisfactory distinctness. We regret that this is not true of the plates of the brain. These are all too small to exhibit with any clearness the fissures and convolutions, etc. True these are often indistinct on the actual brain, and our work stands for actual nature, but one large photograph of a brain, with these features fairly developed, would be much better than the two or three small ones which fail to show satisfactorily the important landmarks of the cerebral surface. But the majority of the plates are excellent and well indexed.

As to the text, the most important feature of it, the description of anatomy by *regions*, rather than a division of the book into sections of muscles, arteries, nerves, etc., must commend itself to every teacher of anatomy. It is *regional* anatomy which the surgeon encounters, and it should be the duty of teachers and the books to furnish the student with that sort of knowledge which will be of the greatest practical value. We have been unable to give this book as thorough an examination as we wished, but we have been much pleased with it and think that Dr. McClellan has reason to be proud of his work. Some errors are to de found, here and there. On page 9, "Extra-and intra-venous circulation," should be *extra-cranial* and *intra-cranial venous*, etc. On page 26 it is said that the left vertebral artery has an independent origin from the arch of the aorta. It is stated, page 222, that the renal vein is the only vein below the diaphragm which lies in front of its corresponding artery. Both the superior mesenteric and the profunda femoris do the same.

Withal a most excellent work, with little to justify unfavorable criticism. The publishers have presented it in a most attractive style, and the execution of their task leaves nothing to be desired. L. B. G.

---

THE PRINCIPLES AND PRACTICE OF MEDICINE. By William Osler, M. D., formerly Professor of the Institutes of Medicine, McGill University, Montreal, and Professor of Clinical Medicine in the University of Pennsylvania; Professor of Medicine in the Johns Hopkins University, and Physician-in-Chief to the Johns Hopkins Hospital, Baltimore. D. Appleton & Co., New York.

Perhaps no one could bring to the task of preparing a text-book on the practice of medicine a richer experience and a wider range of scientific knowledge than Professor Osler, and his completed work fulfills all that might have been expected from the able and distinguished author. This is the newest book now accessible to the student or practitioner, with a new arrangement of subject-matter and with the newer and more rational ideas of pathology and modes of treatment. The classification in some respects is original. Under specific infectious diseases we find Tuberculosis Malarial Fever and Dysentery. The section on Tuberculosis is very thorough and exhaustive in all forms and phases of the disease. Dr. Osler has expended much time and labor in investigating the nature and causation of malaria, and his results and latest views are fully given. It may possibly be objected that the author is now and then too dogmatic. His opinions are expressed clearly and his style is pleasing and forcible. Speaking of the treatment of anæmia, he says: "The indications are simply three—plenty of food, an open air life and iron." The Loeffler bacillus he regards as the "etiological criterion by which true diphtheria may be distinguished from the other forms of pseudo-membranous inflammation." This work has been furnished forth largely from the extensive hospital experience which Dr. Osler has injoyed for many years, and it will be commended heartily by the reviewer and accepted warmly by the student and practitioner. It will also rank as a text-book in our schools along with the familiar works of Flint and Bartholow and Loomis.

---

THE second meeting of the International Dermatological Congress will be held in Vienna from the 5th to the 10th of September, 1892.

Many of the most distinguished representatives of Dermatology and Syphilography from all countries have promised to present papers, and the indications are that the meeting will be a great success from a scientific standpoint.

The Committee on Organization, through the President, Prof. Kaposi, has extended a cordial invitation to the members of the American Dermatological Association, and of the New York Dermatological Society, and others interested in Dermatology in this country to be present.

The membership fee (five dollars) should be sent, with titles of papers intended for presentation, to the Secretary for North America, Dr. Prince A. Morrow, 66 West 40th street, New York, or to the Secretary-General of the Congress, Dr. Gustav Riehl, Wien $\frac{1}{20}$, Bellaria Strasse 12.

